# A nomogram combining clinical factors and biomarkers for predicting the recurrence of high-risk cutaneous squamous cell carcinoma

**DOI:** 10.1186/s12885-022-10213-2

**Published:** 2022-11-03

**Authors:** Yeongjoo Oh, Zhenlong Zheng, Ki-Yeol Kim, Xiangshu Xu, Meiling Pei, Byungho Oh, Sang Kyem Kim, Kee Yang Chung, Mi Ryung Roh

**Affiliations:** 1 Department of Dermatology, Yonsei University College of Medicine, Yongin Severance Hospital, Yongin, Korea; 2 Department of Dermatology, Yanbian University Hospital, Yanji City, Jilin Provence China; 3 Department of Dermatology and Cutaneous Biology Research Institute, Severance Hospital, Yonsei University College of Medicine, Seoul, Korea; 4 Department of Dental Education, BK21 PLuS Project, Yonsei University College of Dentistry, Seoul, Korea; 5 Department of Pathology, Severance Hospital, Yonsei University College of Medicine, Seoul, Korea; 6 Department of Dermatology, Gangnam Severance Hospital, Cutaneous Biology Research Institute, Yonsei University College of Medicine, 63 Gil 20 Eonju-Ro, Gangnam-Gu, Seoul, 06229 Korea

**Keywords:** Cutaneous squamous cell carcinoma, Clinical risk factors, Biomarkers, Combined risk factors, Nomogram, Prognosis

## Abstract

**Background:**

Although determining the recurrence of cutaneous squamous cell carcinoma (cSCC) is important, currently suggested systems and single biomarkers have limited power for predicting recurrence.

**Objective:**

In this study, combinations of clinical factors and biomarkers were adapted into a nomogram to construct a powerful risk prediction model.

**Methods:**

The study included 145 cSCC patients treated with Mohs micrographic surgery. Clinical factors were reviewed, and immunohistochemistry was performed using tumor tissue samples. A nomogram was constructed by combining meaningful clinical factors and protein markers.

**Results:**

Among the various factors, four clinical factors (tumor size, organ transplantation history, poor differentiation, and invasion into subcutaneous fat) and two biomarkers (Axin2 and p53) were selected and combined into a nomogram. The concordance index (C-index) of the nomogram for predicting recurrence was 0.809, which was higher than that for the American Joint Committee on Cancer (AJCC) 7th, AJCC 8th, Brigham and Women’s Hospital, and Breuninger staging systems in the patient data set.

**Conclusion:**

A nomogram model that included both clinical factors and biomarkers was much more powerful than previous systems for predicting cSCC recurrence.

**Supplementary Information:**

The online version contains supplementary material available at 10.1186/s12885-022-10213-2.

## Background

Cutaneous squamous cell carcinoma (cSCC) is the second most common skin cancer, and its incidence is increasing annually [1, 2]. Complete surgical excision is the treatment of choice for cSCC, and further systemic treatment is not required for localized cSCC [3–7]. However, cSCC often recurs even 1after complete surgical excision, and the prognosis of recurrent cSCC is notably poorer than that of the primary tumor [8, 9]. In a recent study, the cost of inpatient care was much higher for cSCC than for non-cSCC ($66,841 per cSCC patient vs. $37,102 per non-cSCC patient), especially for patients with aggressive cSCC, with recurrence or metastasis [10].

According to previous studies, various clinical parameters, such as age, tumor location on auricle or lip, organ recipient, tumor diameter > 2 cm, poor differentiation, invasion into subcutaneous fat, and presence of perineural invasion are well-known factors for a poor prognosis of cSCC [11–17]. Various clinical staging systems or definitions for high-risk cSCC have also been suggested based on these clinical risk factors [11–16, 18, 19]. However, all currently suggested systems or definitions have limited ability for predicting recurrence [18, 20], and there is a need to identify biomarkers that more accurately predict high-risk cSCC.

Aberrant expressions of various proteins have been found during cSCC progression. In our previous study, we found axis inhibition protein 2 (Axin2), Snail, and melanoma-associated antigen A12 (MAGEA12) to be poor prognostic markers for cSCC [21, 22]. Furthermore, we investigated several candidate biomarkers and validated the value of these proteins for predicting cSCC recurrence.

Since cSCC recurrence develops in a multistage process through the accumulation of alterations in various factors, prediction of the risk of cSCC recurrence using a single factor is difficult. Therefore, by combining several biomarkers and further combining them with clinical factors for synergistic effects, cSCC recurrence can be more accurately predicted. Moreover, a nomogram, which is a mathematical formula constructed using various factors with different weights, is another tool for predicting cancer prognosis, and it reflects complex factors.

In this retrospective study, we aimed to evaluate the predictive value of clinical factors and biomarkers in cSCC recurrence and to construct a powerful risk prediction model for cSCC recurrence using combined factors applied in a nomogram.

## Materials and methods

### Patient selection

Patients with cSCC who underwent Mohs micrographic surgery at the Department of Dermatology, Severance Hospital, Seoul, South Korea from 2000 to 2017 were retrospectively reviewed. This study was approved by the Institutional Review Board for Bioethics of Yonsei University Health System, Severance Hospital (4–2018-0331).

### Clinical factors

We selected several candidate factors using previous literature on cSCC risk prediction. Clinical factors such as age, sex, tumor location, organ transplantation history, tumor size, differentiation grade, and invasion depth were analyzed. These candidate factors were considered in the patient cohort, and the association of individual factors with recurrence was analyzed. Various clinical factors were then combined to investigate the best clinical factor combinations for predicting recurrence, and predictive ability was analyzed for each combination.

### Biomarkers and immunohistochemistry

For biomarker evaluation, tumor tissues from 145 patients stored in the Department of Pathology were retrospectively reviewed. Formalin-fixed paraffin-embedded tissue samples were cut into 4-μm tissue sections for immunohistochemistry. Antigen retrieval and blocking of endogenous peroxidase activity were performed after deparaffinization and rehydration of tissue sections.

In our previous study, we selected genes that were involved in important biological processes of cancer cells, such as epithelial-mesenchymal transition (EMT), formation of functional invadopodia, and tumor suppression. Further, we evaluated the biological functions and clinicopathological significance of the genes in cSCC [21, 22]. In the present study, we validated the clinicopathologic significance of previously selected candidate biomarkers and further investigated tumor suppressor gene proteins such as p53, p16, AT-rich interaction domain (ARID) 1A, and ARID1B as candidate prognostic markers using the cSCC cohort. Cortactin (CTTN, Abcam Inc., Cambridge, MA, USA), pTyr421-CTTN (LifeSpan BioSciences Inc., Seattle, WA, USA), pTyr466-CTTN (LifeSpan BioSciences Inc.), MAGEA12 (Abcam Inc.), p21 (Abcam Inc.), p16 (Dako Inc., Glostrup, Denmark), p53 (Dako Inc.), ARID1A (Abcam Inc.), ARID1B (Abcam Inc.), Axin2 (Abcam Inc.), and Snail (Abcam Inc.) primary antibodies were used for this study. Tissue sections were incubated with primary antibodies at room temperature for 1 h. REAL EnVision HRP Rabbit/Mouse Detection System (Dako Inc.) was used as a secondary antibody. After visualizing with 3,3′-diaminobenzidine (Dako Inc.), counterstaining was performed with hematoxylin (Abcam Inc.). Histoscores for each protein expression were calculated with the weighted histoscore method according to the tissue staining intensity and percentage of positive cells [23]. Patients were subdivided into two groups: low (histoscore 0–100) and high (histoscore 101–300) expression.

### Statistical analysis

The effects of various factors on the prediction of cSCC recurrence were assessed with the Cox proportional-hazards model. Various types of metrics were suggested in previous studies to evaluate the prediction models, such as the concordance index (C-index) and the integrated discrimination improvement index [24–29]. The selection of the most suitable metrics helps create a powerful prediction model. In accordance with previous studies [24–27], the recurrence prediction ability was calculated using the C-index for different combinations of clinical factors. The protein expression in each group of patients was compared using the chi-square test. Kaplan–Meier analysis and log-rank test were used to evaluate the survival rate between groups in our cohort. The predictive nomogram was created using a combination of clinical factors and biomarkers for cSCC recurrence and evaluated using the C-index. The American Joint Committee on Cancer (AJCC) 7th, AJCC 8th, Brigham and Women’s Hospital (BWH), and Breuninger staging systems for cSCC were applied to the patient data set, and the C-index for predicting recurrence was calculated for each staging system [14, 15, 19]. SPSS for Windows version 23.0 (IBM Corp., Armonk, NY, USA) and R (R Foundation for Statistical Computing, Vienna, Austria) were used for the statistical analysis in this study.

## Results

### Patient demographics in the cSCC cohort

The cSCC cohort comprised 145 patients (70 men, 75 women; median age: 75 years; age range: 30–98 years) with cSCC (mean follow-up period: 22 months) treated with Mohs micrographic surgery. Of these, 20 (13.8%) patients showed recurrence, and 125 (86.2%) patients did not show recurrence during follow-up. Among the 20 patients with recurrence in our cohort, 12 patient showed only local recurrence, 3 patients showed both local and distant recurrence, and 5 patients had regional nodal recurrence or distant metastasis.

**Fig. 1:**
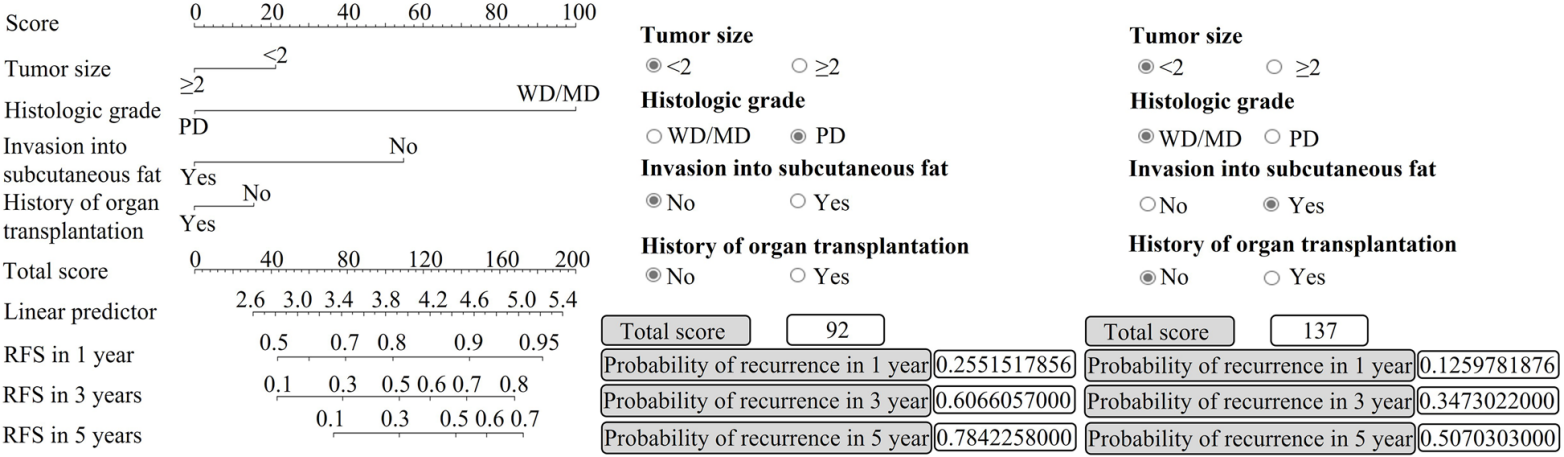
Predictive nomogram including four clinical factors (C-index 0.650 in our patient cohort). RFS: recurrence-free survival; WD: well-differentiated; MD: moderately differentiated; PD: poorly differentiated

The clinicopathological characteristics of the patients are described in Table 1. As shown in Table 1, no significant association was found between any clinicopathological factor and recurrence. We then combined different clinical factors and explored the predictive accuracies (Supplementary Fig. 1). The C-index was the highest when four clinicopathological factors were combined, and among various combinations of four clinicopathological factors, tumor size, histologic grade, invasion depth, and organ transplantation history showed the highest predictive accuracy (Supplementary Table 1). When a nomogram was constructed using these clinical factors, the C-index of the combined clinical factors for predicting cSCC recurrence was approximately 0.650 (Fig. 1).

**Table 1:** Demographics of 145 cSCC patients

Variables	Total (n)	Recurred cases n (%)	*p*-value*	HR (95% CI)
Total cases	145	20 (13.79)		
Mean age, years	73.66	68.7	0.589	0.990 (0.957–1.026)
Sex, n				
Male	70	4 (5.71)	1	
Female	75	16 (21.33)	0.285	0.601 (0.236–1.529)
Location, n				
Head and neck	121	17 (14.05)	1	
Trunk	2	0 (0)	0.986	0.000 (0.000–0.000)
Extremities	2	0 (0)	0.996	0.000 (0.000–0.000)
Acral	20	3 (15.00)	0.978	1.018 (0.293–3.533)
Organ transplantation				
No	135	17 (12.59)	1	
Yes	10	3 (30.00)	0.699	1.281 (0.365–4.499)
Tumor size (mean, cm)				
≤ 2 cm	92	10 (10.87)	1	
> 2 cm	53	10 (18.87)	0.091	2.147(0.884–5.214)
Differentiation grade				
WD	68	11 (16.18)	1	
MD	66	6 (0.09)	0.692	0.800 (0.266–2.407)
PD	11	3 (27.27)	0.058	3.653 (0.955–13.971)
Invasion of subcutaneous fat				
Absent	126	16 (12.70)	1	
Present	19	4 (21.05)	0.126	2.407 (0.782–7.412)

^*^ *p*-values were calculated using univariate cox regression analysis; *cSCC* Cutaneous Squamous Cell Carcinoma, *HR* Hazard ratio, *CI* Confidence Interval, *WD* Well differentiated, *MD* Moderately differentiated, *PD* Poorly differentiated

### Characteristics of protein expression for the candidate biomarkers in cSCC

**Fig. 2:**
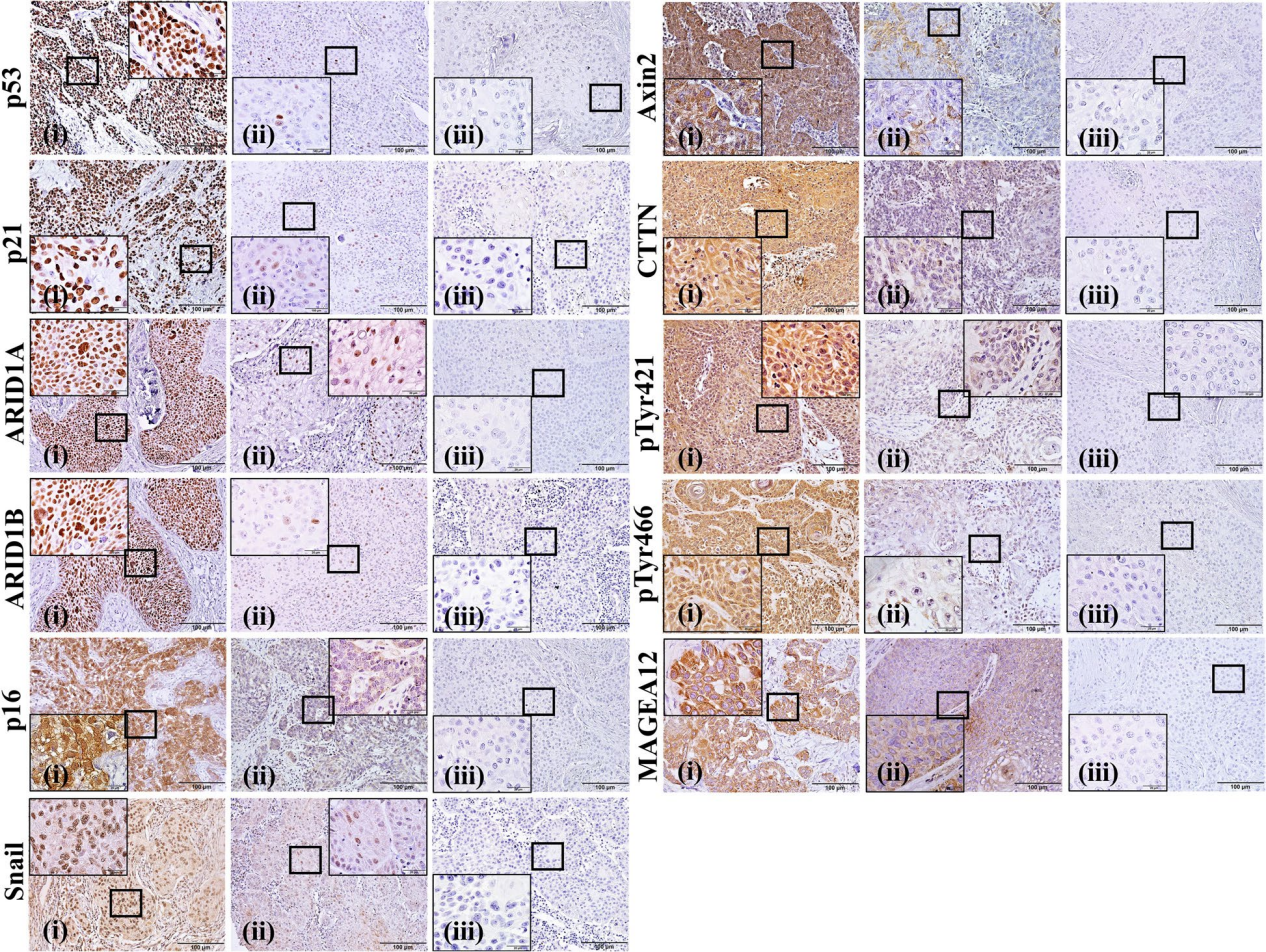
Expression pattern of biomarkers in the cSCC tissue samples. Each column shows high (i), low (ii), and negative (iii) expression. Each row shows the expression patterns of different biomarkers. p53, p21, ARID1A, and ARID1B showed nuclear expression pattern in cSCC cells, and p16 and SNAIL showed expression in both the nucleus and cytoplasm. Axin2, CTTN, pTyr421-CTTN, pTyr466-CTTN, and MAGEA12, showed cytoplasmic expression (original magnification: × 200, scale bar: 100 μm; magnification for inset micrograph: × 1000, scale bar: 20 μm). ARID: AT-rich interaction domain; Axin2: axis inhibition protein 2; cSCC: cutaneous squamous cell carcinoma; MAGEA12: melanoma-associated antigen A12

The candidate biomarkers showed various expression patterns (Fig. 2). cSCC cells typically showed nuclear expression of p53, p21, ARID1A, and ARID1B. Cytoplasmic expressions of Axin2, CTTN, pTyr421-CTTN, pTyr466-CTTN, and MAGEA12 were also found. In addition, p16 and Snail expressions were detected in both the nucleus and cytoplasm of cSCC cells. The differences between patients with or without recurrence in tissue immunoreactivity against candidate biomarkers are shown in Supplementary Table 2. Kaplan–Meier analysis showed that the immunoreactivity of p53, ARID1A, Axin2, pTyr421-CTTN, pTyr466-CTTN, MAGEA12, p16, and Snail were significantly related to the recurrence-free survival of cSCC patients (Supplementary Fig. 2).

#### Selection of combined risk factors for predicting cSCC recurrence

**Fig. 3:**
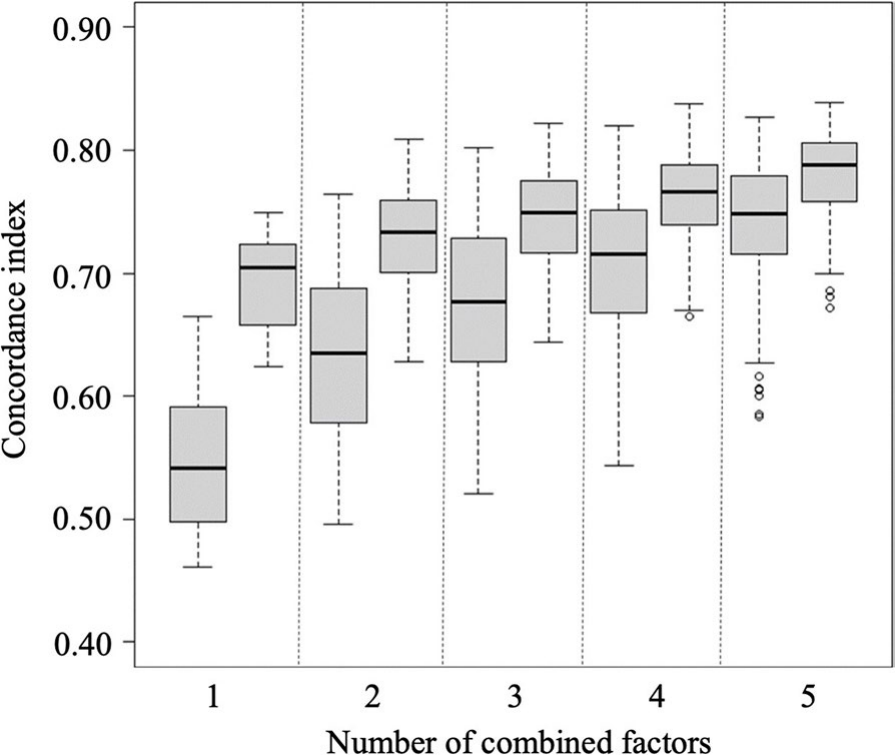
Concordance index (C-index) according to number of combined protein factors. The C-indexes increased as the number of factors increased. First boxplot: only protein(s); Second boxplot: protein(s) and clinicopathologic factors including tumor size, histologic grade, invasion into subcutaneous fat, and history of organ transplantation

To implement an optimal prediction model, we first attempted to identify the number of biomarkers that could show the most efficient predictive power when combined with clinical factors. To identify the most valuable biomarker set, we combined different proteins and explored their predictive ability (Fig. 3). C-indexes increased as the number of biomarkers increased. For ease of clinical application, we identified the number of biomarkers that showed the highest predictive power with the smallest number of proteins, and the most optimal result was obtained with two proteins. The C-index achieved by combining selected clinicopathological factors and biomarkers is shown in Supplementary Table 3.

#### Nomogram construction using combined risk factors

**Fig. 4:**
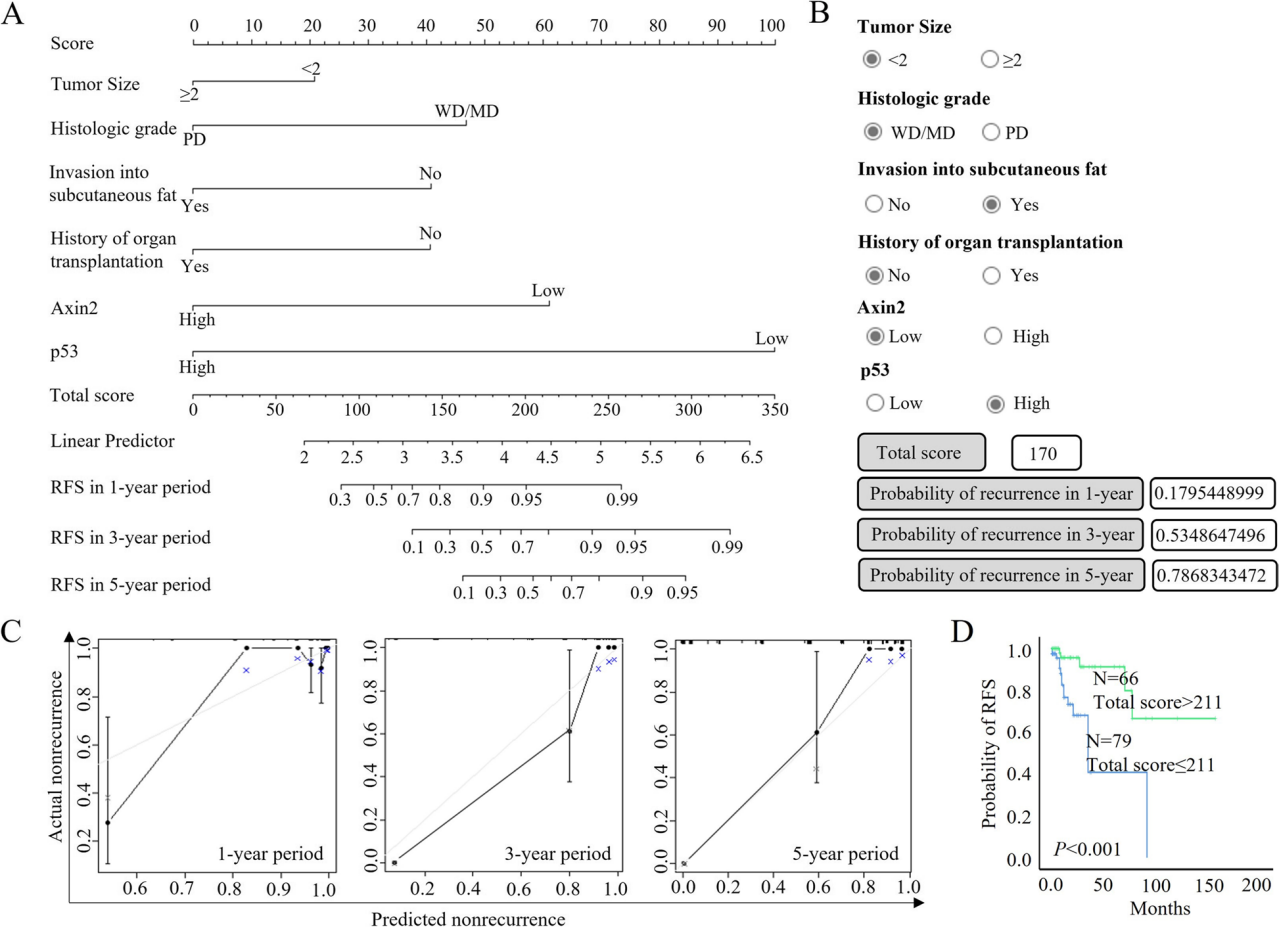
Nomogram constructed by combining clinical risk factors and biologic markers. **A** A diagram of the constructed nomogram **B** HTML construct of the nomogram. The probability of recurrence in 1, 3, and 5 years can be calculated by checking the status of patients. **C** Calibration plots of the nomogram for predicting the probability recurrence in 1, 3, and 5 years. **D** Kaplan–Meier graph showing recurrence-free survival according to score at nomogram. RFS: recurrence-free survival

We constructed a nomogram (Fig. 4A) to predict the probability of recurrence by combining data concerning tumor size, histologic grade, subcutaneous invasion, history of organ transplantation, and Axin2 and p53 expressions. The C-index of the nomogram was approximately 0.809 (Supplementary Table 3). The probability of recurrence in 1, 3, and 5 years was determined by the calculated total scores. For comparison with the traditional staging systems, four staging systems, namely AJCC 7th, AJCC 8th, BWH, and Breuninger, were considered in our patient set, and their C-indices were 0.6264, 0.7073, 0.7086, and 0.7185, respectively, which were lower than the C-index of our model (Supplementary Table 4) [14, 15, 19].

For practical usage of the nomogram, a hypertext markup language (HTML) format of the nomogram was also constructed (Fig. 4B). In the HTML format, the probability of recurrence in 1, 3, and 5 years was calculated by checking the status of patients. For example, a patient with tumor size < 2 cm, well to moderately differentiated histologic grade, invasions of tumor cells in subcutaneous fat, no history of organ transplantation, low expression of Axin2, and high expression of p53 would have a total score of 170, which would correspond to a probability of recurrence of 18.0%, 53.5%, and 78.7% in 1, 3, and 5 years, respectively.

A statistically significant relationship between recurrence predicted by the nomogram and actual outcomes of cSCC patients was found in this study. When the prediction values calculated by nomogram were assigned to the x-axis and the actual clinical outcomes were assigned to the y-axis, the calibration plot showed a solid line in each indicated follow-up period (Fig. 4C). We also evaluated whether the total points calculated by the nomogram could be used to predict recurrence in our cohort. The total points ranged from 68 to 311, and the patients were further divided into two groups using the median value of the total point calculated by nomogram (cutoff score: 211). We found a significant difference in recurrence-free survival between the two groups of patients, according to Kaplan–Meier analysis (Log rank test, *p* < 0.001) (Fig. 4D). These findings suggest that the prediction model for the recurrence of cSCC, constructed using combined factors, is ideal.

## Discussion

Predicting recurrence and identifying high-risk cSCC patients that require strict surveillance are major issues in cSCC management. The incidence of basal cell carcinoma is higher, but it shows a much indolent prognosis. The prognosis of melanoma is much poorer; however, there is a well-established clinical staging system for melanoma as well as crucial target biomarkers such as BRAF. In contrast, cSCC, which is the second most common skin cancer following basal cell carcinoma, has no clinically effective biomarkers or a staging system with a high predictive value.

Consistent with previous observations, traditional staging systems that utilize only clinical factors did not show a satisfactory ability in predicting recurrence in our cohort. No significant association was found between patient outcome and the classic clinical prognostic predictors such as age, tumor location, history of organ transplantation, histologic grade, and invasion depth in our cohort. These discrepancies might be due to the fact that predictive power of the single clinical factors is not high enough to apply to any cohort. Because of the limited predictive power of clinical factors, predictive biomarkers have been investigated for risk stratification of SCC. In a recent study, Shapanis et al. conducted proteomic profiling on cSCC tissues and compared the metastasis and non-metastasis groups [30]. In their study, the prediction model incorporating significant proteins showed higher predictive power for metastasis compared with previous clinical staging systems in the cSCC patient group, which implies that protein expressions are important factors and should be included in a risk prediction model.

In our previous study, Axin2, Snail, pTyr421-CTTN, pTyr466-CTTN, and MAGEA12 expressions were significantly associated with recurrence-free survival [21, 22]. In the current study, tumor suppressor genes, such as ARID1A, p53, and p16 were also found to be risk factors for cSCC recurrence in our cohort (Supplementary Fig. 2).

For higher predictive power, combinations of biomarkers were included in the nomogram. Although the predictive power increased with a higher number of protein combinations, two biomarkers were selected, considering clinical practicality. When combined with clinical factors, p53 and Axin2 showed the highest synergistic power and were included in the final nomogram.

In response to several types of stress, the tumor suppressor protein, p53, can be stabilized in the nucleus and acts as a transcription factor for many genes that are involved in the proliferation and differentiation of normal cells. In contrast, in various cancer cells, especially for ultraviolet light-induced carcinogenesis, mutation of the p53 gene is the most common genetic alteration during progression, which often leads to the overexpression of the p53 protein [31–33]. The association of p53 overexpression with SCC prognosis has been consistently reported and was noted in our study as well [34, 35].

Axin2, as a component of the β-catenin degradation complex under the absence of Wnt signals, was initially identified as a tumor suppressor gene [36]. However, recent studies have demonstrated the oncogenic activities of Axin2 in both premalignant lesions and various malignancies [37, 38]. Studies showed that the abundant expression of Axin2 was significantly associated with the malignant transformation of oral leukoplakia, and both Wnt signaling and the invasive ability of cancer cells were attenuated by Axin2 knockdown in colorectal cancers [37–40]. Moreover, as a downstream target of the Wnt signaling pathway, Axin2 acts as a nucleocytoplasmic shuttle of glycogen synthase kinase 3 (GSK-3), thereby inhibiting GSK-3-dependent Snail degradation and resulting in the nuclear stabilization of Snail, which is a key mediator of EMT [41]. Consistent with previous studies [21, 41], Axin2 expression was significantly associated with Snail expression, and increased Axin2 expression was also a significant risk factor for recurrence, in our cohort. Moreover, some investigators showed that microRNA-34, a transcriptional target of p53, can suppress Axin2 expression due to its binding with the 5’ and 3’ untranslated regions of Axin2 [42]. Considering the inhibitory effect of p53 on Axin2 expression, genetic alteration of p53 may be a sign of increased Axin2 expression. Therefore, the synergistic effect of p53 and Axin2 expression may result from the critical role of EMT mediated via the p53-Axin2-GSK3-Snail axis in cSCC progression.

A nomogram, which is a mathematical formula with a diagram, has recently been adopted in the field of oncology [43]. The nomogram may provide patients with personalized predictions and aid in determining treatment plans. Disparate risk factors for each cancer type can be mathematically weighted and combined into a nomogram as a reliable and reproducible prediction model. To date, various nomograms have been used for the prediction of various cancers [44–50]. However, there are only a few reports on the application of the nomogram in cSCC. In the present study, we constructed a predictive nomogram to determine the probability of recurrence in 1, 3, and 5 years in cSCC patients after Mohs micrographic surgery.

This study had certain limitations. Some important clinical factors such as perineural invasion and vascular invasion were not included in this study because of missing entries in > 60% of the patients. Moreover, there may be other crucial predictive protein markers or genetic alterations related to cSCC recurrence that were not included in our nomogram. Nevertheless, in the current prediction model, we found that the C-index was approximately 0.65 when only clinical factors were considered, but this increased to approximately 0.809 when the clinical factors were combined with p53 and Axin2 expression. In addition, the calibrated nomogram showed high predictive accuracy, in our study.

The combined expressions of the p53 and Axin2 proteins may be useful for assessing the risk of cSCC recurrence. Despite the lack of external validation, the nomogram constructed by combining appropriate clinical risk factors and biomarkers showed higher predictive value than previous prediction systems for cSCC. By using a relevant nomogram model, high-risk cSCC patients can be identified more accurately, and stricter surveillance would be possible in these patients.

## Supplementary Information

**Additional file 1.**

## Data Availability

The datasets used and analyzed during the current study are available from the corresponding author on reasonable request.

## References

[1] Lomas A, Leonardi-Bee J, Bath-Hextall F (2012). A systematic review of worldwide incidence of nonmelanoma skin cancer. Br J Dermatol.

[2] Stang A, Khil L, Kajuter H, Pandeya N, Schmults CD, Ruiz ES, Karia PS, Green AC (2019). Incidence and mortality for cutaneous squamous cell carcinoma: comparison across three continents. J Eur Acad Dermatol Venereol.

[3] Kauvar AN, Arpey CJ, Hruza G, Olbricht SM, Bennett R, Mahmoud BH (2015). Consensus for nonmelanoma skin cancer treatment, Part II: Squamous cell carcinoma, including a cost analysis of treatment methods. Dermatol Surg.

[4] van Lee CB, Roorda BM, Wakkee M, Voorham Q, Mooyaart AL, de Vijlder HC, Nijsten T, van den Bos RR (2019). Recurrence rates of cutaneous squamous cell carcinoma of the head and neck after Mohs micrographic surgery vs. standard excision: a retrospective cohort study. Br J Dermatol.

[5] Marrazzo G, Zitelli JA, Brodland D (2019). Clinical outcomes in high-risk squamous cell carcinoma patients treated with Mohs micrographic surgery alone. J Am Acad Dermatol.

[6] Leibovitch I, Huilgol SC, Selva D, Hill D, Richards S, Paver R (2005). Cutaneous squamous cell carcinoma treated with Mohs micrographic surgery in Australia II. Perineural invasion J Am Acad Dermatol.

[7] Stratigos AJ, Garbe C, Dessinioti C, Lebbe C, Bataille V, Bastholt L, Dreno B, Concetta Fargnoli M, Forsea AM, Frenard C (2020). European interdisciplinary guideline on invasive squamous cell carcinoma of the skin: Part 2. Treatment Eur J Cancer.

[8] Oh Y, Kim J, Zheng Z, Kim SK, Chung KY, Roh MR (2020). Risk factors for recurrence in cutaneous squamous cell carcinoma after Mohs micrographic surgery: a retrospective review of 237 Asian patients. J Dermatol.

[9] Dean NR, Sweeny L, Magnuson JS, Carroll WR, Robinson D, Desmond RA, Rosenthal EL (2011). Outcomes of recurrent head and neck cutaneous squamous cell carcinoma. J Skin Cancer.

[10] Tripathi R, Knusel KD, Ezaldein HH, Bordeaux JS, Scott JF (2020). Characteristics of patients hospitalized for cutaneous squamous cell carcinoma. Dermatol Surg.

[11] Baum CL, Wright AC, Martinez JC, Arpey CJ, Brewer JD, Roenigk RK, Otley CC (2018). A new evidence-based risk stratification system for cutaneous squamous cell carcinoma into low, intermediate, and high risk groups with implications for management. J Am Acad Dermatol.

[12] Thompson AK, Kelley BF, Prokop LJ, Murad MH, Baum CL (2016). Risk factors for cutaneous squamous cell carcinoma recurrence, metastasis, and disease-specific death: a systematic review and meta-analysis. JAMA Dermatol.

[13] Farasat S, Yu SS, Neel VA, Nehal KS, Lardaro T, Mihm MC, Byrd DR, Balch CM, Califano JA, Chuang AY (2011). A new American joint committee on cancer staging system for cutaneous squamous cell carcinoma: creation and rationale for inclusion of tumor (T) characteristics. J Am Acad Dermatol.

[14] Lydiatt WM, Patel SG, O’Sullivan B, Brandwein MS, Ridge JA, Migliacci JC, Loomis AM, Shah JP (2017). Head and Neck cancers-major changes in the American Joint Committee on cancer eighth edition cancer staging manual. CA Cancer J Clin.

[15] Karia PS, Jambusaria-Pahlajani A, Harrington DP, Murphy GF, Qureshi AA, Schmults CD (2014). Evaluation of American Joint Committee on Cancer, International Union Against Cancer, and Brigham and Women's Hospital tumor staging for cutaneous squamous cell carcinoma. J Clin Oncol.

[16] Stratigos AJ, Garbe C, Dessinioti C, Lebbe C, Bataille V, Bastholt L, Dreno B, Fargnoli MC, Forsea AM, Frenard C (2020). European interdisciplinary guideline on invasive squamous cell carcinoma of the skin: Part 1. epidemiology, diagnostics and prevention. Eur J Cancer.

[17] Squamous cell skin cancer (Version 1.2021). External link https://www.nccn.org/professionals/physician_gls/pdf/squamous.pdf

[18] Ruiz ES, Karia PS, Besaw R, Schmults CD (2019). Performance of the American Joint Committee on Cancer staging manual, 8th edition vs the Brigham and Women's Hospital Tumor Classification System for cutaneous squamous cell carcinoma. JAMA Dermatol.

[19] Breuninger H, Brantsch K, Eigentler T, Hafner HM (2012). Comparison and evaluation of the current staging of cutaneous carcinomas. J Dtsch Dermatol Ges.

[20] Roscher I, Falk RS, Vos L, Clausen OPF, Helsing P, Gjersvik P, Robsahm TE (2018). Validating 4 staging systems for cutaneous squamous cell carcinoma using population-based data: a nested case-control study. JAMA Dermatol.

[21] Zhao G, Kim KY, Zheng Z, Oh Y, Yoo DS, Lee ME, Chung KY, Roh MR, Jin Z (2020). AXIN2 and SNAIL expression predict the risk of recurrence in cutaneous squamous cell carcinoma after Mohs micrographic surgery. Oncol Lett.

[22] Zhao G, Bae JY, Zheng Z, Park HS, Chung KY, Roh MR, Jin Z (2019). Overexpression and implications of melanoma-associated antigen A12 in pathogenesis of human cutaneous squamous cell carcinoma. Anticancer Res.

[23] Zhang X, Zheng Z, Shin YK, Kim KY, Rha SY, Noh SH, Chung HC, Jeung HC (2014). Angiogenic factor thymidine phosphorylase associates with angiogenesis and lymphangiogenesis in the intestinal-type gastric cancer. Pathology.

[24] Harrell FE, Califf RM, Pryor DB, Lee KL, Rosati RA (1982). Evaluating the yield of medical tests. JAMA.

[25] Lubsen J, Pool J, van der Does E (1978). A practical device for the application of a diagnostic or prognostic function. Methods Inf Med.

[26] Kattan MW, Shariat SF, Andrews B, Zhu K, Canto E, Matsumoto K, Muramoto M, Scardino PT, Ohori M, Wheeler TM (2003). The addition of interleukin-6 soluble receptor and transforming growth factor beta1 improves a preoperative nomogram for predicting biochemical progression in patients with clinically localized prostate cancer. J Clin Oncol.

[27] Kattan MW, Karpeh MS, Mazumdar M, Brennan MF (2003). Postoperative nomogram for disease-specific survival after an R0 resection for gastric carcinoma. J Clin Oncol.

[28] Kerr KF, McClelland RL, Brown ER, Lumley T (2011). Evaluating the incremental value of new biomarkers with integrated discrimination improvement. Am J Epidemiol.

[29] Pencina MJ, D'Agostino RB, Demler OV (2012). Novel metrics for evaluating improvement in discrimination: net reclassification and integrated discrimination improvement for normal variables and nested models. Stat Med.

[30] Shapanis A, Lai C, Smith S, Coltart G, Sommerlad M, Schofield J, Parkinson E, Skipp P, Healy E (2021). Identification of proteins associated with development of metastasis from cutaneous squamous cell carcinomas (cSCCs) via proteomic analysis of primary cSCCs. Br J Dermatol.

[31] Oren M (2003). Decision making by p53: life, death and cancer. Cell Death Differ.

[32] Bennett WP, Hussain SP, Vahakangas KH, Khan MA, Shields PG, Harris CC (1999). Molecular epidemiology of human cancer risk: gene-environment interactions and p53 mutation spectrum in human lung cancer. J Pathol.

[33] Missero C, Antonini D (2014). Crosstalk among p53 family members in cutaneous carcinoma. Exp Dermatol.

[34] Florence ME, Massuda JY, Soares TC, Stelini RF, Poppe LM, Brocker EB, Metze K, Cintra ML, de Souza EM (2015). p53 immunoexpression in stepwise progression of cutaneous squamous cell carcinoma and correlation with angiogenesis and cellular proliferation. Pathol Res Pract.

[35] Campos MA, Macedo S, Fernandes MS, Pestana A, Pardal J, Batista R, Vinagre J, Sanches A, Baptista A, Lopes JM (2020). Prognostic significance of RAS mutations and P53 expression in cutaneous squamous cell carcinomas. Genes (Basel).

[36] Polakis P (2002). Casein kinase 1: a Wnt'er of disconnect. Curr Biol.

[37] Wu ZQ, Brabletz T, Fearon E, Willis AL, Hu CY, Li XY, Weiss SJ (2012). Canonical Wnt suppressor, Axin2, promotes colon carcinoma oncogenic activity. Proc Natl Acad Sci U S A.

[38] Major MB, Roberts BS, Berndt JD, Marine S, Anastas J, Chung N, Ferrer M, Yi X, Stoick-Cooper CL, von Haller PD (2008). New regulators of Wnt/beta-catenin signaling revealed by integrative molecular screening. Sci Signal..

[39] Ahn SY, Kim NH, Lee K, Cha YH, Yang JH, Cha SY, Cho ES, Lee Y, Cha JS, Cho HS (2017). Niclosamide is a potential therapeutic for familial adenomatosis polyposis by disrupting Axin-GSK3 interaction. Oncotarget.

[40] Zhang X, Kim KY, Zheng Z, Kim HS, Cha IH, Yook JI (2017). Snail and Axin2 expression predict the malignant transformation of oral leukoplakia. Oral Oncol.

[41] Yook JI, Li XY, Ota I, Hu C, Kim HS, Kim NH, Cha SY, Ryu JK, Choi YJ, Kim J (2006). A Wnt-Axin2-GSK3beta cascade regulates Snail1 activity in breast cancer cells. Nat Cell Biol.

[42] Kim NH, Cha YH, Kang SE, Lee Y, Lee I, Cha SY, Ryu JK, Na JM, Park C, Yoon HG (2013). p53 regulates nuclear GSK-3 levels through miR-34-mediated Axin2 suppression in colorectal cancer cells. Cell Cycle.

[43] Iasonos A, Schrag D, Raj GV, Panageas KS (2008). How to build and interpret a nomogram for cancer prognosis. J Clin Oncol.

[44] Balachandran VP, Gonen M, Smith JJ, DeMatteo RP (2015). Nomograms in oncology: more than meets the eye. Lancet Oncol.

[45] Bertolli E, de Macedo MP, Calsavara VF, Pinto CAL, Duprat Neto JP (2019). A nomogram to identify high-risk melanoma patients with a negative sentinel lymph node biopsy. J Am Acad Dermatol.

[46] Jeong SH, Kim RB, Park SY, Park J, Jung EJ, Ju YT, Jeong CY, Park M, Ko GH, Song DH (2020). Nomogram for predicting gastric cancer recurrence using biomarker gene expression. Eur J Surg Oncol.

[47] Rocco B, Sighinolfi MC, Sandri M, Puliatti S, Bianchi G (2018). A novel nomogram for predicting ECE of prostate cancer. BJU Int.

[48] Su J, Miao LF, Ye XH, Cui MS, He XF (2019). Development of prognostic signature and nomogram for patients with breast cancer. Medicine (Baltimore).

[49] Wang S, Yang L, Ci B, Maclean M, Gerber DE, Xiao G, Xie Y (2018). Development and validation of a nomogram prognostic model for SCLC patients. J Thorac Oncol.

[50] Wang SJ, Patel SG, Shah JP, Goldstein DP, Irish JC, Carvalho AL, Kowalski LP, Lockhart JL, Holland JM, Gross ND (2013). An oral cavity carcinoma nomogram to predict benefit of adjuvant radiotherapy. JAMA Otolaryngol Head Neck Surg.

