# Supplementary material for: A nomogram combining clinical factors and biomarkers for predicting the recurrence of high-risk cutaneous squamous cell carcinoma
Source: BMC Cancer. 2022 Nov 3;22:1126. doi: 10.1186/s12885-022-10213-2 (PMC9632077; doi:10.1186/s12885-022-10213-2)
Supplement: Supplementary file 1 — Additional file 1. [file 12885_2022_10213_MOESM1_ESM.docx]

**Supplementary Material**

**Supplementary Tables**

Table S1. Predictive accuracies of the combination of randomly selected four clinicopathologic factors.

| Factor 1 | Factor 2 | Factor 3 | Factor 4 | C-index |
| --- | --- | --- | --- | --- |
| Invasion depth | Size | Histologic grade | Transplantation | 0.650439367 |
| Sex | Invasion depth | Transplantation | Histologic grade | 0.622079038 |
| Transplantation | Histologic grade | Age | Invasion depth | 0.620790378 |
| Location | Invasion depth | Transplantation | Size | 0.615289982 |
| Age | Invasion depth | Histologic grade | Size | 0.612295082 |
| Invasion depth | Transplantation | Size | Sex | 0.610896309 |
| Sex | Size | Histologic grade | Invasion depth | 0.605327869 |
| Invasion depth | Location | Transplantation | Histologic grade | 0.605326461 |
| Transplantation | Sex | Age | Invasion depth | 0.604037801 |
| Location | Transplantation | Sex | Invasion depth | 0.599742268 |
| Age | Invasion depth | Size | Transplantation | 0.599472759 |
| Location | Histologic grade | Invasion depth | Age | 0.598877306 |
| Location | Histologic grade | Invasion depth | Size | 0.597131148 |
| Location | Histologic grade | Transplantation | Age | 0.585996564 |
| Sex | Age | Invasion depth | Histologic grade | 0.580032077 |
| Sex | Location | Invasion depth | Histologic grade | 0.574017642 |
| Sex | Transplantation | Age | Location | 0.573539519 |
| Size | Sex | Invasion depth | Age | 0.572950821 |
| Size | Histologic grade | Location | Transplantation | 0.570035149 |
| Age | Location | Invasion depth | Transplantation | 0.569673541 |
| Age | Invasion depth | Sex | Location | 0.569607057 |
| Size | Invasion depth | Location | Sex | 0.568852459 |
| Age | Invasion depth | Location | Size | 0.568032787 |
| Transplantation | Age | Histologic grade | Size | 0.563884007 |
| Transplantation | Histologic grade | Age | Sex | 0.563230241 |
| Size | Histologic grade | Sex | Location | 0.561475411 |
| Location | Size | Age | Histologic grade | 0.560655738 |
| Sex | Histologic grade | Transplantation | Size | 0.560369069 |
| Sex | Size | Age | Histologic grade | 0.559426231 |
| Transplantation | Size | Age | Location | 0.557293497 |
| Size | Location | Transplantation | Sex | 0.550263621 |
| Histologic grade | Sex | Transplantation | Location | 0.549054983 |
| Sex | Size | Location | Age | 0.540163934 |
| Size | Age | Sex | Transplantation | 0.540158172 |
| Sex | Histologic grade | Location | Age | 0.509061748 |

Table S2. Association between protein expression and cSCC recurrence

| Variables | Total, n (%) | Recurrence, n (%) | | *p-*value* |
| --- | --- | --- | --- | --- |
|  |  | No | Yes |  |
| Total samples | 145 (100) | 125 (86.2) | 20 (13.8) |  |
| p53 |  |  |  |  |
| Low | 67 (46.2) | 63 (94.0) | 4 (6.0) | 0.015 |
| High | 78 (53.8%) | 62 (79.5) | 16 (20.5%) |  |
| p21 |  |  |  |  |
| Low | 87 (60) | 72 (82.8) | 15 (17.2) | 0.218 |
| High | 58 (40) | 53 (91.4) | 5 (8.6) |  |
| ARID1A |  |  |  |  |
| Low | 83 (57.2) | 69 (83.1) | 14 (16.9) | 0.235 |
| High | 62 (42.8) | 56 (90.3) | 6 (9.7) |  |
| ARID1B |  |  |  |  |
| Low | 80 (55.2) | 67 (83.8) | 13 (16.3) | 0.469 |
| High | 65 (44.8) | 58 (89.2) | 7 (10.8) |  |
| Axin2 |  |  |  |  |
| Low | 99 (68.3) | 90 (90.9) | 9 (9.1) | 0.021 |
| High | 46 (31.7) | 35 (76.1) | 11 (23.9) |  |
| CTTN |  |  |  |  |
| Low | 50 (34.5) | 46 (92.0) | 4 (8.0) | 0.205 |
| High | 95 (65.5) | 79 (83.2) | 16 (16.8) |  |
| pTyr421-CTTN |  |  |  |  |
| Low | 64 (44.1) | 57 (89.1) | 7 (10.9) | 0.47 |
| High | 81 (55.9) | 68 (84.0) | 13 (16.0) |  |
| pTyr466-CTTN |  |  |  |  |
| Low | 72 (49.7) | 66 (91.7) | 6 (8.3) | 0.09 |
| High | 73 (50.3) | 59 (80.8) | 14 (19.2) |  |
| MAGEA12 |  |  |  |  |
| Low | 85 (58.6) | 77 (90.6) | 8 (9.4) | 0.088 |
| High | 60 (41.4) | 48 (80.0) | 12 (20.0) |  |
| Snail |  |  |  |  |
| Low | 92 (63.4) | 84 (91.3) | 8 (8.7) | 0.025 |
| High | 53 (36.6) | 41 (77.4) | 12 (22.6) |  |
| p16 |  |  |  |  |
| Low | 27 (18.6) | 26 (96.3) | 1 (3.7) | 0.124 |
| High | 118 (81.4) | 99 (83.9) | 19 (16.1) |  |

* Fisher’s exact test; ARID, AT-Rich interaction domain; Axin2, axis inhibition protein 2; MAGEA12; Melanoma-associated Antigen A12

Table S3. Predictive accuracies of the combination of two randomly selected proteins plus clinicopathological factors (tumor size, histologic grade, invasion depth, and organ transplantation history)

| Protein 1 | Protein 2 | C-index* |
| --- | --- | --- |
| P53 | Axin2 | 0.808602151 |
| Snail | Axin2 | 0.786648746 |
| Axin2 | P16 | 0.784856631 |
| pTyr421-CTTN | Axin2 | 0.783064516 |
| P53 | Snail | 0.779032258 |
| P53 | pTyr466-CTTN | 0.772759857 |
| P21 | Axin2 | 0.772311828 |
| MAGEA12 | Axin2 | 0.771863799 |
| P16 | P53 | 0.768727599 |
| pTyr466-CTTN | P16 | 0.765143369 |
| P16 | Snail | 0.758870968 |
| pTyr421-CTTN | P53 | 0.75797491 |
| Axin2 | ARID1A | 0.752598566 |
| pTyr466-CTTN | Snail | 0.749014337 |
| ARID1B | Axin2 | 0.744086022 |
| Snail | MAGEA12 | 0.743637993 |
| P53 | ARID1B | 0.742741935 |
| P53 | CTTN | 0.741845878 |
| MAGEA12 | P16 | 0.739605735 |
| P21 | P53 | 0.736917563 |
| CTTN | pTyr466-CTTN | 0.736469534 |
| Snail | ARID1A | 0.736021505 |
| pTyr466-CTTN | P21 | 0.734677419 |
| Snail | P21 | 0.734229391 |
| pTyr421-CTTN | P16 | 0.733333333 |
| CTTN | Snail | 0.726164875 |
| P53 | ARID1A | 0.725716846 |
| P16 | P21 | 0.72078853 |
| MAGEA12 | pTyr466-CTTN | 0.719444444 |
| ARID1B | Snail | 0.716756272 |
| pTyr466-CTTN | ARID1A | 0.714964158 |
| MAGEA12 | pTyr421-CTTN | 0.713620072 |
| ARID1B | pTyr466-CTTN | 0.706899642 |
| CTTN | P16 | 0.706451613 |
| CTTN | MAGEA12 | 0.705555556 |
| MAGEA12 | P21 | 0.70421147 |
| P16 | ARID1B | 0.701075269 |
| ARID1A | MAGEA12 | 0.70062724 |
| P21 | pTyr421-CTTN | 0.698387097 |
| CTTN | pTyr421-CTTN | 0.690770609 |
| MAGEA12 | ARID1B | 0.688082437 |
| pTyr421-CTTN | ARID1A | 0.67688172 |
| P16 | ARID1A | 0.674641577 |
| ARID1B | P21 | 0.66702509 |
| P21 | CTTN | 0.666129032 |
| ARID1B | pTyr421-CTTN | 0.661200717 |
| ARID1B | CTTN | 0.655824373 |
| ARID1B | ARID1A | 0.65 |
| ARID1A | P21 | 0.649551971 |
| CTTN | ARID1A | 0.628494624 |

*C-index for a combination of selected clinicopathological factors with biomarkers

The highest accuracy was achieved by combining the selected clinicopathological factors with Axin2 and p53.

Table S4. C-indices of previous staging systems and our nomogram

|  | **C-index** |
| --- | --- |
| Combined factors in this study | 0.8086 |
| AJCC 7^th^ edition | 0.6264 |
| AJCC 8^th^ edition | 0.7073 |
| BWH | 0.7086 |
| Breuninger | 0.7185 |

AJCC, American Joint Committee on Cancer; BWH, Brigham and Women’s Hospital

**Supplementary Figures**


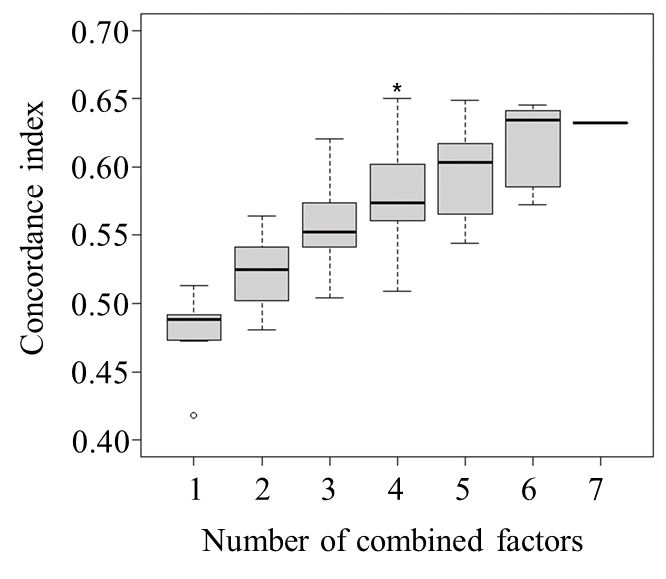


**Supplementary Figure 1**. Concordance index (C-index) according to the number of combined clinical factors. The C-index was highest when four clinical factors were combined. (*)


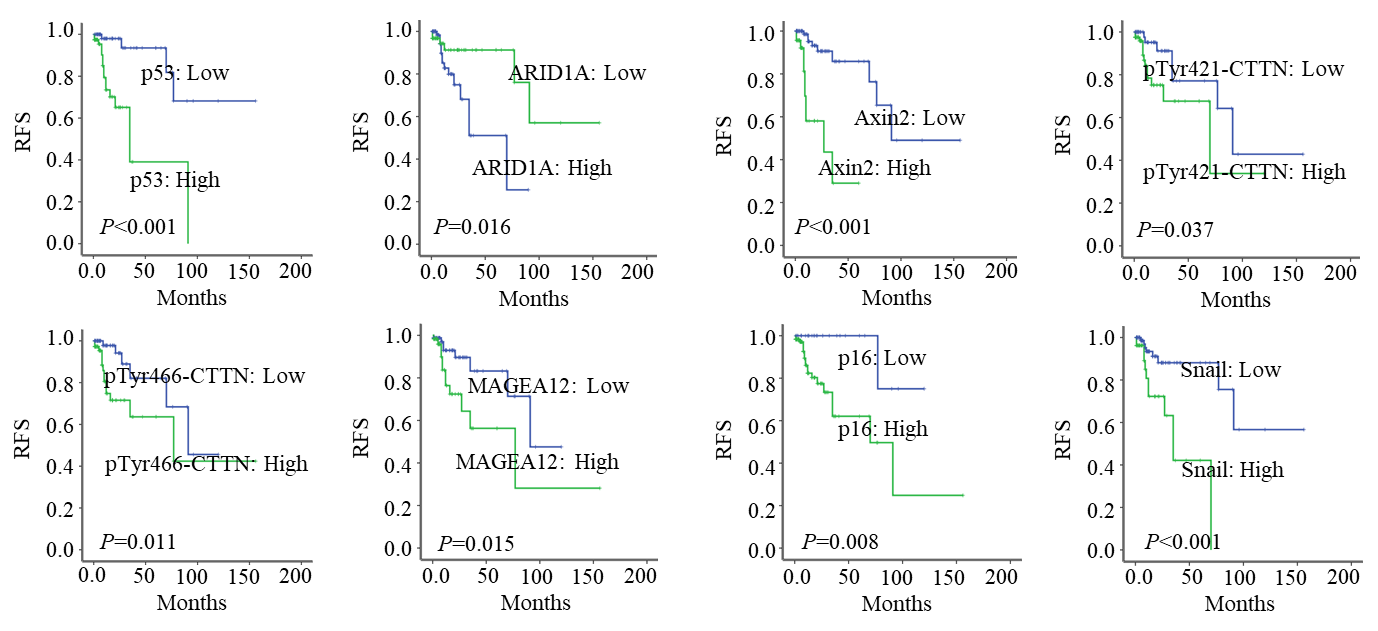


**Supplementary Figure 2**. Kaplan-Meier graph showing recurrence-free survival (RFS) according to the expression of each protein marker. The immunoreactivities of p53, ARID1A, Axin2, pTyr421-CTTN, pTyr466-CTTN, MAGEA12, p16, and Snail were significantly related to the RFS of cSCC patients in our cohort.
